# Autonomic Dysfunction and Postural Orthostatic Tachycardia Syndrome Secondary to Abuse

**DOI:** 10.1016/j.jaccas.2025.105773

**Published:** 2025-11-26

**Authors:** Samuel Reiss, Rachel Reiss, Craig Reiss

**Affiliations:** Department of Cardiovascular Medicine, St Luke’s University, Chesterfield, Missouri, USA

**Keywords:** awareness, cardiac risk, cardiovascular disease, chest pain, electrophysiology, lifestyle, palpitations, risk factor, tachycardia, treatment

## Abstract

Postural orthostatic tachycardia syndrome (POTS) and other forms of autonomic dysfunction are increasingly recognized in patients with histories of physical or psychological trauma. Emerging evidence suggests that trauma-related dysregulation of the autonomic nervous system may manifest as POTS, with hypervigilance and hyperadrenergic states proposed as potential mechanisms. However, the relationship between sexual abuse and subsequent autonomic dysfunction remains unclear. We present a series of patients referred to cardiology for suspected POTS, highlighting clinical features, potential mechanisms, and treatment considerations. These findings emphasize the need for trauma-informed evaluation to improve patient care and guide future research.

Postural orthostatic tachycardia syndrome (POTS) is a type of dysautonomia characterized by an excessive increase in heart rate upon standing, without orthostatic hypotension. Common symptoms include fatigue, lightheadedness, palpitations, gastrointestinal complaints, and cognitive difficulties.^1^, ^2^, ^3^

**Take-Home Messages**

• Both patients and providers are becoming increasingly aware of the entities of postural orthostatic tachycardia syndrome and autonomic dysfunction.
• These patients are frequently referred to cardiologists to aid in their diagnosis and management.
• It is critical to realize that many of these patients may have experienced significant sexual trauma and recognizing and referring patients for appropriate therapy is crucial for their treatment and overall well-being.

Recent epidemiological data indicate that POTS affects approximately 0.2% to 1% of the population, typically beginning in adolescence or early adulthood, with a marked female predominance.^4^ This demographic overlap with groups at high risk for trauma raises important questions about causality and comorbidity.

Abuse and trauma, especially when chronic or severe, can disrupt the hypothalamic-pituitary-adrenal (HPA) axis and sympathetic nervous system, which can cause long-term autonomic instability.^5^ Despite this link, the connection between trauma and autonomic dysfunction, including POTS, has received limited attention in clinical practice. This case series aims to address this gap, highlight potential mechanisms, and inform patient care by documenting patients with autonomic dysfunction and POTS who report histories of abuse.

## Methods

Patients presenting with symptoms of inappropriate sinus tachycardia, orthostatic hypotension, and POTS based on established criteria (eg, a sustained increase in heart rate of ≥30 beats/min within 10 minutes of standing or head-up tilt, without orthostatic hypotension) were included. Detailed histories were obtained, including triggers, comorbidities, and psychosocial factors, with particular attention paid to abuse histories (physical, emotional, or sexual). Autonomic testing, laboratory evaluations, and psychosocial assessments were performed as indicated.

## Case 1

A 20-year-old woman with a history of hypermobile Ehlers-Danlos syndrome, seizure disorder, and POTS presented to the cardiology clinic for evaluation. Her POTS symptoms began at age 11, with her first fainting episode occurring at age 12 and progressive decline occurring through adolescence. By age 17, she had difficulty walking for more than 15 minutes outdoors; in college, climbing stairs raised her heart rate to 160 beats/min, and she cited her symptoms as the reason she withdrew after 1 semester. A positive tilt-table test result at 19 confirmed POTS. In the clinic, she disclosed that she had experienced psychological trauma during puberty and was sexually abused by peers in high school.

## Case 2

A 33-year-old woman with a medical history of Ehlers-Danlos syndrome, atrioventricular nodal re-entrant tachycardia status postablation, fibromyalgia, irritable bowel syndrome, and migraines was referred to the cardiology clinic for evaluation and management of POTS.

Since adolescence, she had experienced near-blackouts upon standing, and she had to walk rather than run the mile during school sports. She was initially diagnosed with vasovagal syncope. Her symptoms worsened in 2016, requiring medications, including midodrine and pindolol, salt/fluid loading, and the implantation of a loop recorder, which led to a diagnosis of POTS. She remains very limited in her activities and reports spending most of her time in bed or at rest.

Her orthostatic vitals in the clinic did not show a significant drop in her blood pressure. Her heart rate increased by 25 beats after 3 minutes of standing, and further checks were discontinued because the patient began feeling unwell.

She disclosed having experienced sexual assault at age 16. Her school did not pursue the matter, and many did not believe her, adding an aspect of psychological trauma. She failed to connect to a trauma counselor at the time of the incident and has not sought subsequent therapy.

## Case 3

A 29-year-old woman with seropositive rheumatoid arthritis and major depressive disorder presented with tachycardia and presyncope, especially during showers, with heart rates up to 168 beats/min on monitoring. At rest, her heart rate was approximately 100 beats/min, rising to 130 to 140 beats/min with minimal exertion. She reported marked heat sensitivity and difficulty remaining upright for long periods. Over the past 5 years, she lost 100 pounds, recently with tirzepatide, and she was prescribed PRN metoprolol by a prior physician.

Trauma history included sexual abuse at age 8 years by a relative, at age 16 years by a boyfriend, and at age 17 years by another individual. She also experienced the death of her mother at age 17 years and subsequent verbal abuse by her stepmother, leading to homelessness in 2020.

## Case 4

A 38-year-old woman with longstanding anxiety and depression was referred to the cardiology clinic by her psychiatrist for concerns of POTS. She reported persistently elevated heart rates since childhood and a syncopal episode at age 16, requiring hospitalization. In adulthood, 3 complicated deliveries and COVID-19 in 2022 worsened symptoms, causing labile blood pressure, tachycardia, and exertional dyspnea. Cardiac imaging revealed a transiently reduced ejection fraction that later normalized. Monitoring showed sinus tachycardia up to 160 beats/min, with an average rate of 108 beats/min. After her July 2023 delivery, she developed intermittent orthostatic dizziness and racing heart rates. She was diagnosed with POTS, and her evaluation for pheochromocytoma showed negative results.

She disclosed having experienced sexual trauma between the ages of 12 and 16, for which she underwent counseling.

At the time of referral, she had started on beta blockers and begun POTS-specific physical therapy. She has noted significant symptom improvement with continued counseling, physical therapy, and oral fluid and salt resuscitation.

## Case 5

A 21-year-old nursing student with a history of anorexia nervosa requiring prior hospitalization was referred to the clinic for recurrent monthly syncope episodes and concerns of POTS. Six to eight years ago, she had begun experiencing episodes of lightheadedness, tunnel vision, and fainting while standing. Her orthostatic vitals showed a heart rate rise from 83 to 108 beats/min and a modest blood pressure drop from 126/80 to 104/89 mm Hg, suggesting autonomic dysfunction though not meeting the criteria for POTS.

Her trauma history included severe sexual, physical, and emotional abuse between ages 5 and 16, including sexual abuse at age 12 by a family member. She attends weekly trauma counseling.

## Case 6

A 54-year-old woman with a history of anxiety, an eating disorder, and celiac disease presented with recurrent episodes of fatigue, palpitations, and dyspnea. She also reported intermittent episodes of diaphoresis, nausea, and tachycardia up to 160 beats/min with minimal exertion, as well as a syncopal event. She was referred to electrophysiology, and tilt-table testing revealed tachycardia with mild hypotension, suggestive of a vasodepressive component of the syncope. She also had documented episodes of bradycardia, autonomic dysfunction, and inappropriate sinus tachycardia but did not meet full criteria for POTS.

The patient and her twin brother were sexually abused by their adoptive father from ages 5 to 18 years, during which time he also abused several of her friends. She has undergone cognitive behavioral therapy.

## Case 7

A 32-year-old woman with a history of rheumatoid arthritis, Sjӧgren syndrome, Hashimoto thyroiditis, and hypermobile Ehlers-Danlos syndrome presented to the cardiology clinic for a second opinion regarding dysautonomia and palpitations.

She completed 2 tilt-table tests: one triggered vasovagal syncope after treatment with provocative medication, and the other was not typical for POTS but suggestive of dysautonomia.

She reported complex post-traumatic stress disorder after repeated rape by peers at age 14 and severe emotional, verbal, and physical abuse by her brother. After these events, she attempted suicide, engaged in substance use, and underwent prolonged counseling.

## Results

All patients were referred to cardiology for palpitations and tachycardia, or autonomic dysfunction, often with a presumptive POTS diagnosis. Several patients also reported orthostatic intolerance and chronic fatigue. Emotional symptoms such as anxiety, depression, and hypervigilance were common. Ultimately, all patients were diagnosed with autonomic dysfunction, most frequently within the context of POTS. A majority of them disclosed histories of sexual abuse during adolescence or early adulthood, with many female patients experiencing sexual abuse even earlier. In many cases, this disclosure occurred only after trust was established through repeated visits.

Abuse histories varied but commonly involved prolonged exposure to harmful sexual, emotional, or physical environments. Temporal links were observed between the onset of abuse, symptom worsening, and POTS development. Treatment options varied but included hydration with salt loading, compression devices, physical rehabilitation, medications, and trauma-focused psychotherapy.

## Discussion

This case series suggests an association between trauma, particularly sexual abuse, and autonomic dysregulation, including POTS. Patients often presented with cardiovascular symptoms such as palpitations or syncope but without structural heart disease. Instead, dysautonomia symptoms frequently reflected unresolved psychological trauma. In this series, all patients had a history of psychological trauma before the onset of dysautonomia symptoms, highlighting a connection between abuse and autonomic dysfunction.

Several mechanisms may underlie this link: chronic activation of the HPA axis, altered catecholamine sensitivity, and maladaptive neuroplasticity.^6^^,^^7^ Early-life stress has been found to alter brain structures involved in autonomic regulation, including the insula, anterior cingulate cortex, and amygdala, leading to dysautonomia. Shared pathophysiology between dysautonomia and trauma-related conditions such as post-traumatic stress disorder may further account for the overlapping symptoms in affected individuals.

Recognition of this connection between abuse and autonomic dysfunction has important clinical implications. Trauma-informed care is essential to address both the physiological and psychological contributors of these conditions. Future research should clarify the biological basis of this relationship and evaluate integrated treatment models.

## Conclusions

It is essential to note that, in many cases, the cardiologist was the first health care provider to uncover histories of abuse, highlighting the importance of trauma-informed evaluation in patients with autonomic dysfunction. Recognizing these histories is crucial for accurate diagnosis, referral to psychiatric and psychological support, and comprehensive treatment. A multidisciplinary approach, incorporating cardiology, physical therapy, and trauma therapy, is critical, and further research should investigate this complex relationship and develop targeted interventions.

## Funding Support and Author Disclosures

The authors have reported that they have no relationships relevant to the contents of this paper to disclose.
